# Photography and Murder

**Published:** 1863-09

**Authors:** 


### PHOTOGRAPHY AND MURDER.

Under this singular and ominous title an absurd correspondence has been going the round of the journals, and has been accepted in some quarters as conveying solemn truth of serious import. A Mr. Warner, photographer, on reading an account of the murder of Emma Jackson in St. Giles's, addressed a letter to Detective Officer James F. Thompson, informing him that "if the eyes of a murdered person be photographed within a certain time after death, upon the retina will be found depicted the last thing that appeared before them, and that in the present case the features of the murderer would most probably be found thereon." The writer exemplified his statement by the fact of his having, four years ago, taken a negative of the eye of a calf a few hours after death, and, upon a microscopic examination of the same, found depicted thereon the lines of the pavement on the slaughter-house floor. This negative is unfortunately broken, and the pieces lost. Mr. Warner states his opinion that the subject is of too great importance and interest to be passed heedlessly by, because if the fact was known through the length and breadth of the land, it would, in his estimation, tend materially to decrease that most horrible of all crimes—murder.

Mr. Thompson, superintendent of detectives, replies in similarly solemn style, capping the marvellous information of his correspondent by a detail of circumstantial accuracy. He says: "The secret you convey in your letter—photographing the eyes of a murdered person—is one of the greatest importance, but unfortunately it is unavailing in this instance, for various reasons, three of which I will give you: 1st, life had been extinct some forty hours prior to my seeing the body of Emma Jackson; 2d, the eyes were closed; 3d, a post-mortem examination has been made, and she has been buried—shell coffin—since Monday last. In conversing with an eminent oculist some four years ago upon this subject, I learned that unless the eyes were photographed within twenty-four hours after death no result would be obtained, the object transfixed thereon vanishing in the same manner as undeveloped negative photograph exposed to light. I did not therefore resort to this expedient."

The multitude of reasons given by the sapient superintendent of detectives for not attempting an absurd impossibility will remind his readers of the forty reasons of the mayor for the town-gunner not firing a salute, of which the first—namely, the absence of powder—was held to be sufficient. The information derived from the eminent oculist is singularly interesting. But, before attempting the photographic feat which is suggested, Mr. Thompson might find useful practice in endeavoring to subtract the sound of a flute from a ton of coals, or to draw out the moonshine from cucumber seeds. *Quid vetat ridendo dicerc verum.* Mr. Warner has hoaxed himself, and the superintendent of detectives takes the name of oculist in vain. "Stone walls do not a prison make," and the bars on Mr. Warner's photograph were not akin to the pavement of the slaughter-house. Mr. Thompson may assure Sir Richard Mayne that such a photograph taken more than twenty-four hours after death will succeed as well as if taken two minutes after—and no better.—*London Lancet.*

